# Increased deficit of visual attention span with development in Chinese children with developmental dyslexia

**DOI:** 10.1038/s41598-018-21578-5

**Published:** 2018-02-16

**Authors:** Jing Zhao, Menglian Liu, Hanlong Liu, Chen Huang

**Affiliations:** Key Laboratory of Learning and Cognition, School of Psychology, Capital Normal University, Beijing, BJ 100089 China

## Abstract

It has been suggested that orthographic transparency and age changes may affect the relationship between visual attention span (VAS) deficit and reading difficulty. The present study explored the developmental trend of VAS in children with developmental dyslexia (DD) in Chinese, a logographic language with a deep orthography. Fifty-seven Chinese children with DD and fifty-four age-matched normal readers participated. The visual 1-back task was adopted to examine VAS. Phonological and morphological awareness tests, and reading tests in single-character and sentence levels were used for reading skill measurements. Results showed that only high graders with dyslexia exhibited lower accuracy than the controls in the VAS task, revealing an increased VAS deficit with development in the dyslexics. Moreover, the developmental trajectory analyses demonstrated that the dyslexics seemed to exhibit an atypical but not delayed pattern in their VAS development as compared to the controls. A correlation analysis indicated that VAS was only associated with morphological awareness for dyslexic readers in high grades. Further regression analysis showed that VAS skills and morphological awareness made separate and significant contributions to single-character reading for high grader with dyslexia. These findings suggested a developmental increasing trend in the relationship between VAS skills and reading (dis)ability in Chinese.

## Introduction

Developmental dyslexia (DD) is a specific impairment in learning how to read accurately, fluently and in gaining reading comprehension, which is not accounted for by problems in general intelligence, learning opportunities, general motivation, or sensory acuity^1^. It has been suggested that DD is caused by multiple genetic, cognitive, and environmental risk factors^2^. Based on the relevant literature in the context of different languages^3,4^, two major frameworks have always been presented for the possible mechanism of reading disability: the linguistic and non-linguistic framework hypotheses. First, the linguistic framework hypothesis postulates that individuals with dyslexia usually exhibit core deficits in phonological processing, orthographic awareness, semantic analysis, and their interactions (e.g., orthographic-to-phonological mapping)^5^. Although the above impairments were consistently reported by research on different languages^6^, it has been suggested that deficits in accessing and manipulating phonological information were more remarkable for dyslexic readers of alphabetic languages^5^, and difficulty with morphological awareness may contribute more greatly to reading disability in Chinese^7^. Second, the non-linguistic framework hypothesis proposes that dysfunction at the linguistic level may stem from more fundamental deficits in basic cognitive processing, including perception^8^, working memory^9^, and visual attention^10^.

Given that dyslexic readers often tend to transpose letters or characters (e.g., “was” for “saw” in English reading; “”[pronounced/xue2 xi2/, meaning study; the number refers to tone; the same is shown below] for “”[/xi2 xue2/, non-word] in Chinese reading) and exhibit visual confusion (e.g., characters/letters appear to blur and move around in their eyes) when they are trying to read^11^, and reading begins with visual processing, some researchers have thus indicated that the exploration of dyslexia should be traced back to the basic visual-spatial processing underlying the language skills. Some relevant studies have focused on the visual attention span (VAS) of dyslexics^10^. VAS refers to the amount of distinct visual elements that can be processed in parallel in a multi-element array^10,12^. Previous studies typically adopted whole/partial report tasks and relevant modified paradigms to measure the VAS ability^10^. According to the visual attention span deficit hypothesis proposed by Bosse *et al*.^8^, VAS impairment might contribute to poor reading outcomes in dyslexics independent of their phonological disorders.

In the context of alphabetic languages, several studies reported VAS dysfunction in some dyslexic readers from both behavioral and neural aspects^10,13–20^. The relevant literature found poor behavioral performance (including lower accuracy, longer reaction times, and poor task sensitivity) through whole/partial report tasks in both French and Portuguese children with dyslexia as compared to their age-matched controls^13–16,19^, in which the orthographic depths of French and Portuguese were deep and intermediate, respectively^21^. Neuroimaging findings revealed that French children and adults with dyslexia both exhibited lower brain activation in their parietal region (e.g., bilateral superior parietal lobule, left supramarginal gyrus) functioning in the visual attention span, with multiple-element paradigms being modified on the basis of the whole/partial report tasks, such as a visual categorization task^17–20^. Moreover, high frequency repetitive transcranial magnetic stimulation over the bilateral parietal regions of the dyslexic adults improved accuracy of their non-word reading^22^. A case study of a VAS intervention on a French girl with dyslexia reported that the training resulted in the faster identification of words, improved text reading, and increased activation in her bilateral superior parietal cortices and bilateral precuneus^15^. These findings provided evidence for the causal link between the visual attention span deficit and reading disability.

However, some other studies failed to replicate the VAS deficit in dyslexics^23–25^, in which the dyslexic readers did not differ from the non-impaired readers in the VAS-related tasks^23–25^. Hawelka and Wimmer found that German adults with dyslexia showed similar reaction times as normal readers in a partial report task^24^. Yeari *et al*.’s study reported that there were no significant differences in the recognition sensitivity between dyslexic adults in Hebrew and the age-matched skilled readers in either whole or partial report tasks^25^. Because it has been suggested that a potential modulation of the visual attention span on reading depends on orthographic transparency^26^, the above inconsistencies in the existing literature might thus be associated with differences in orthographic depth. Studies reporting VAS impairments in the dyslexic individuals were usually in the context of languages with deep/intermediate orthography (e.g., French), while studies that found an absence of VAS deficit in dyslexics were mostly in languages with shallow orthography (e.g., German and Hebrew). Orthographic transparency may exert an influence on the nature of reading strategies^27^. In transparent orthographies (i.e., languages with shallow orthographies), readers prompt sub-lexical decoding strategies based on the rules of grapheme-to-phoneme correspondence (GPC), with the characterization of small orthographic units during reading. By contrast, in opaque languages (i.e., languages with a deep orthography), the availability of larger orthographic units is more regular than smaller ones, which encourages readers to use whole-word reading strategies through the lexical route^28^. Accordingly, it could be suggested that the VAS skills may be influenced by the orthographic depth. Namely, the visual attention span might contribute more to reading in languages with deep orthographies. A recent cross-language study provided support for the above inference to some extent^26^. Awadh *et al*. recruited Arabic, French and Spanish adults, and they found that the visual attention span of French adults was correlated with their scores on an oral reading fluency test, in which the orthographic depth of French was deeper than that of both Spanish and Arabic. This finding revealed that the visual attention span might exhibit a more significant relation to reading skills in languages with deep orthography^26^. The VAS ability might be better for languages with deep orthography, in which reading is usually involved in processing a larger orthographic unit, compared to that in languages with shallow orthography. Consequently, the VAS impairment of dyslexic readers might be more obviously exhibited in processing languages with deep orthography. Whereas, the VAS may be small for both dyslexics and normal readers in languages with shallow orthography, and group differences may not be markedly presented.

Particularly for languages with deep orthography, another aspect that should be carefully considered is the understanding of age-related changes in the relationship between the visual attention span and reading. With reading development, there is a transition from relying on the sub-lexical decoding strategies of letter-by-letter spelling to depending on a strategy through the whole lexical-word route^29^. The visual attention span may contribute more to a global coarse-grain strategy than a sub-lexical small-grain strategy^30^, and thus it could be inferred that VAS skills might play a more important role during the later stages of reading development, as supported by the findings in the relevant literature^31^. A developmental study by Bosse *et al*. found that the independent contribution of phonological skills to reading was much greater in the 1^st^ grade than in later grades, revealing a developmental decrease, while there seemed to be a developmental increasing trend in the unique contribution of VAS to reading (especially for reading irregular words)^31^. Would the VAS ability of dyslexic readers then vary with their reading development? According to the developmental pattern of the relationship between the visual attention span and reading in the normal readers mentioned above, is it possible that the visual attention span deficit of dyslexics might be more obviously exhibited during the later stages of development, in which a larger visual attention span is required for reading processes (especially for processing the larger orthographic units)? These issues need to be addressed in further studies.

As a logographic writing system, Chinese lacks strict GPC rules and has a deep orthography. In the early stage of reading development in Chinese, beginning readers are taught Chinese primarily through Pinyin, which expresses spoken Chinese using the Roman alphabet and plays a special role in bridging the gap between speech and the written form of Chinese characters^32^. With increased reading experience, the direct connections between the visual forms and the corresponding meanings of Chinese characters would be well established, and the utilization of Pinyin in reading procedures would be gradually diminished^33^. That is, the developmental pattern is a decrease in the influence of phonological skills and a developmental increase in the involvement of morphological processing^32^. Considering that visual words/characters in Chinese directly and globally map onto relevant speech sounds primarily through an addressed route that greatly differs from that in alphabetic languages (an assembled way based on GPC rules)^34^, it is interesting to explore the development of the relationship between the visual attention span and Chinese reading (disability). Is the developmental pattern of the visual attention span in Chinese individuals with dyslexia similar to the prediction based on alphabetic languages with deep orthography? Alternatively, would there be a language specificity for this relationship in Chinese? That is, the VAS of Chinese dyslexic readers would have a relationship to the addressed mapping between whole visual forms of characters and whole syllables during the early stages of Chinese reading development (i.e., through the mediation of Pinyin), considering that the visual-spatial attention has been suggested to be essential in creating the visual images of spoken stimuli^35^. To our knowledge, there has been no relevant developmental study. Reports exploring the relationship between the visual attention span and Chinese reading (disability) are also scarce, and the relevant findings are inconsistent. Mi *et al*.^36^ adopted whole and partial report tasks with verbal responses to measure the visual attention span, in which the target stimuli were five Chinese characters with high frequency. Their results showed that there were no significant differences in the visual attention span between Chinese dyslexic children and age-matched normal readers. However, the participants in their study were selected from the 2^nd^ to 5^th^ grades. The large range of participant ages might be implicated with the additional influence of developmental changes in the visual attention span and its relation to reading in Chinese. Additionally, a recent study^37^ found that Chinese adults exhibited a close relationship between their visual attention span and silent reading fluency, suggesting that the visual attention span might play an important role in Chinese reading even for skilled readers.

The present study investigated the developmental pattern of the visual attention span in Chinese children with developmental dyslexia. The aforementioned studies on VAS^10,12^ generally used a letter-report task. This task requires a verbal response and the use of verbal stimuli, and it thus may additionally tap into linguistic processing (such as visual to phonological mapping) besides visual rapid simultaneous processing. To separate these two processes, the visual 1-back task designed by Lallier *et al*.^38^ was adopted to measure the visual attention span with nonverbal stimuli and no verbal report. The visual attention span ability in each position was analyzed to examine the influence of the spatial location on the VAS. Moreover, Chinese dyslexic children from different age groups were recruited to explore the developmental changes in the relationship between their visual attention span and reading disability.

## Results

### Analyses of the scores from the control task on single-figure recognition

A visual control task on single-figure recognition was adopted to examine whether the participants had sufficient efficiency in identifying an individual figure. A two-way ANOVA was submitted to analyze the correct reaction times, accuracy, and d-prime values in the visual control task, with the group (dyslexics vs. controls) and grade (low, middle, high grades) as the two between-subject factors. First, one-sample Kolmogorov-Smirnov tests were conducted to examine whether the dependent variables of accuracy, correct reaction time, and d-prime values showed normal distributions or not. The results showed no significant effects (ps > 0.1), revealing that the test distributions were (nearly) normal. Levene’s tests of homogeneity of variance were then conducted, and the relevant results showed that the variance in accuracy, correct reaction time, and d-prime values were separately equal (ps > 0.1).

The results of the two-way ANOVAs on any of the three dependent variables showed no significant effects or interactions (ps > 0.1). Table 1 displays the relevant information.

**Table 1:** Descriptive statistics of each conditions.

Measures	Low grade		Middle grade		High grade	
	DD (N = 20)	NR(N = 20)	DD(N = 19)	NR(N = 17)	DD(N = 18)	NR(N = 17)
Chronological age *(years)*	8.88 (0.32)	9.14 (0.27)	10.19 (0.43)	10.22 (0.52)	11.68 (0.49)	11.75 (0.27)
Vocabulary *(standard score)*	967 (298)	1983 (281)	1454 (403)	2486 (359)	2099 (247)	3219(45)
Non-verbal IQ *(standard score)*	34.00 (5.93)	35.00 (4.83)	34.33 (7.24)	36.83 (7.43)	39.00 (5.23)	41.17 (7.36)
Phonological awareness	10.42 (3.90)	14.75 (4.80)	12.13 (4.03)	16.15 (6.53)	16.55 (4.11)	21.50 (3.73)
Morphological awareness	10.14 (1.99)	12.67 (2.18)	12.00 (2.00)	14.17 (2.37)	11.50 (1.72)	14.17 (4.07)
**Reading tests**						
Single character level	138.18(54.93)	146.00(67.09)	107.95(38.70)	156.69(59.12)	150.94(51.97)	207.75(63.27)
Sentence level	0.74 (0.10)	0.85(0.05)	0.75(0.07)	0.82(0.08)	0.79(0.07)	0.86(0.06)
**Visual 1-back task**						
***ACC***						
Position 1	0.52 (0.26)	0.48 (0.22)	0.59 (0.20)	0.62 (0.27)	0.58 (0.21)	0.67 (0.23)
Position 2	0.55 (0.24)	0.49 (0.19)	0.49 (0.21)	0.59 (0.21)	0.58 (0.16)	0.69 (0.14)
Position 3	0.82 (0.16)	0.83 (0.16)	0.72 (0.17)	0.73 (0.19)	0.70 (0.16)	0.74 (0.16)
Position 4	0.57 (0.21)	0.53 (0.18)	0.53 (0.16)	0.51 (0.18)	0.51 (0.19)	0.50 (0.14)
Position 5	0.55 (0.22)	0.49 (0.21)	0.52 (0.19)	0.54 (0.23)	0.46 (0.21)	0.54 (0.21)
***RT*** * (milliseconds)*						
Position 1	1747 (633)	1661 (350)	1486 (371)	1324 (457)	1307 (493)	1107 (386)
Position 2	1704 (601)	1682 (470)	1291 (498)	1376 (592)	1234 (404)	1128 (347)
Position 3	1696 (494)	1510 (418)	1311 (418)	1496 (422)	1285 (485)	1071 (304)
Position 4	1694 (658)	1501 (366)	1413 (358)	1345 (573)	1273 (375)	1217 (361)
Position 5	1761 (563)	1569 (549)	1517 (382)	1466 (363)	1351 (587)	1255 (331)
***d-prime***						
Position 1	0.17 (1.15)	−0.26 (0.88)	0.10 (0.46)	0.39 (0.64)	0.13 (0.70)	0.55 (0.67)
Position 2	0.18 (1.22)	−0.001 (0.78)	−0.09 (0.73)	0.21 (0.47)	0.29 (0.48)	0.55 (0.45)
Position 3	0.40 (1.25)	0.25 (1.04)	0.10 (0.99)	0.38 (0.57)	0.41 (0.55)	0.82 (0.59)
Position 4	0.46 (1.11)	−0.03 (0.76)	0.07 (0.53)	−0.03 (0.63)	0.06 (0.66)	−0.05 (0.61)
Position 5	−0.07 (1.02)	−0.10 (0.92)	−0.10 (0.57)	0.14 (0.80)	−0.15 (1.01)	0.10 (0.54)
**Visual control task**						
*ACC*	0.89 (0.08)	0.94 (0.04)	0.92 (0.09)	0.88 (0.07)	0.97 (0.05)	0.94 (0.06)
*RT (milliseconds)*	996 (278)	874 (238)	959 (160)	768 (149)	710 (273)	640 (139)
d-prime	2.46 (0.55)	2.44 (0.88)	2.68 (0.82)	2.12 (1.06)	2.44 (0.66)	2.44 (0.89)

Note. Standard deviations were shown in the parentheses below every value. Measure units are in the parentheses for each item in the ‘Measures’ column. Non-verbal IQ, the score in Standardized Raven’s Matrices test. Vocabulary, the score in the Character Recognition Measure and Assessment Scale for Primary School Children, revealing the reading ability. ACC, accuracy in the visual 1-back task; RT, correct reaction time in the visual 1-back task. DD, dyslexic readers; NR, normal readers.

### Analyses of the scores from the visual 1-back task

#### Analyses based on the group matching design

Correct reaction times longer than three standard deviations away from the mean were excluded (17 observations, or approximately 0.32% of the total). The remaining data were submitted to further analysis. A three-way mixed ANOVA was submitted to analyze the reaction times, accuracy and d-prime values in the visual 1-back task, with the group (dyslexics vs. controls) and grades (low, middle, high grades) as the between-subject factors and the position (i.e., five positions within a string) as the within-subject factor. The results of one-sample Kolmogorov-Smirnov tests exhibited no significant effects (ps > 0.1), suggesting a normal distribution for all the dependent variables. The results of Levene’s test for the homogeneity of variance showed that the variances were equal for accuracy, correct reaction time, and d-prime values under every condition (ps > 0.1). The sphericity assumption was accepted in ANOVA for correct reaction times, while the sphericity assumption was violated in ANOVA for accuracy and d-prime values, in which the Greenhouse-Gasser correction was applied. Post hoc analyses were conducted in which the relevant p-values were corrected by Bonferroni adjustment.

### Reaction times

The ANOVA results for the correct reaction times showed that the main effect of the grade was significant [F(2, 105) = 13.44, p < 0.001, η^2^ = 0.20]. Post hoc analyses showed that children in the low grade responded more slowly than the children in middle and high grades (ps < 0.05), and children from the middle grade exhibited longer reaction times than the ones in the high grades (p = 0.09, marginally significant). There was no any other significant effect or interaction (ps > 0.1).

### Accuracy

The relevant results for accuracy showed that the main effect of the position was significant [F(4, 420) = 29.08, p < 0.001, η^2^ = 0.22]. Post hoc analyses indicated that the accuracy when targets were presented in the middle position of a string (i.e., the 3^rd^ position within a string) was higher than that in the other four positions (ps < 0.001), and there was no significant difference in the accuracy across the other four positions within a string (ps > 0.1).

The position × grade interaction was significant [F(8, 420) = 3.09, p = 0.002, η^2^ = 0.06]. Simple effect analyses showed that the position effect was significant for all three grades [low grade: F(4, 105) = 20.63, p < 0.001, η^2^ = 0.35; middle grade: F(4, 105) = 7.38, p < 0.001, η^2^ = 0.16; high grade: F(4, 105) = 8.72, p < 0.001, η^2^ = 0.22]. Post hoc analyses demonstrated that for the children in the low grades, the accuracy when the target was presented in the 3^rd^ position within a string was higher than that in the other four positions (ps < 0.05), and there was no significant difference in accuracy across the other four position conditions (ps > 0.1). For the students from the middle grade, the accuracy for the 3^rd^ position within a string was higher than that of the 2^nd^, 4^th^ and 5^th^ positions (ps < 0.01), and the accuracy when the target was presented in the 1^st^ position within a string did not differ from the other four positions (p > 0.1). For the children in high grades, there were no significant differences in the accuracy across the 1^st^, 2^nd^, and 3^rd^ positions (ps > 0.1), or between the 4^th^ and 5^th^ positions within a string (p = 0.99); the accuracy when the target was presented in any of the 1^st^, 2^nd^, and 3^rd^ positions was higher than that in both of the 4^th^ and 5^th^ positions (ps < 0.1). The grade effect was only significant when targets were presented in the 1^st^, 2^nd^, 3^rd^ positions of a string. Post hoc analyses showed that children from the low grade showed higher accuracy than the children in the other two grades (ps < 0.05) under the condition of the 3^rd^ position within a string. The grade effects of the 1^st^ and 2^nd^ positions were similar to one another, in which the children in low grades exhibited lower accuracy than those in the high grades (ps < 0.5), while any other comparisons were not significant (ps > 0.1).

**Figure 1:**
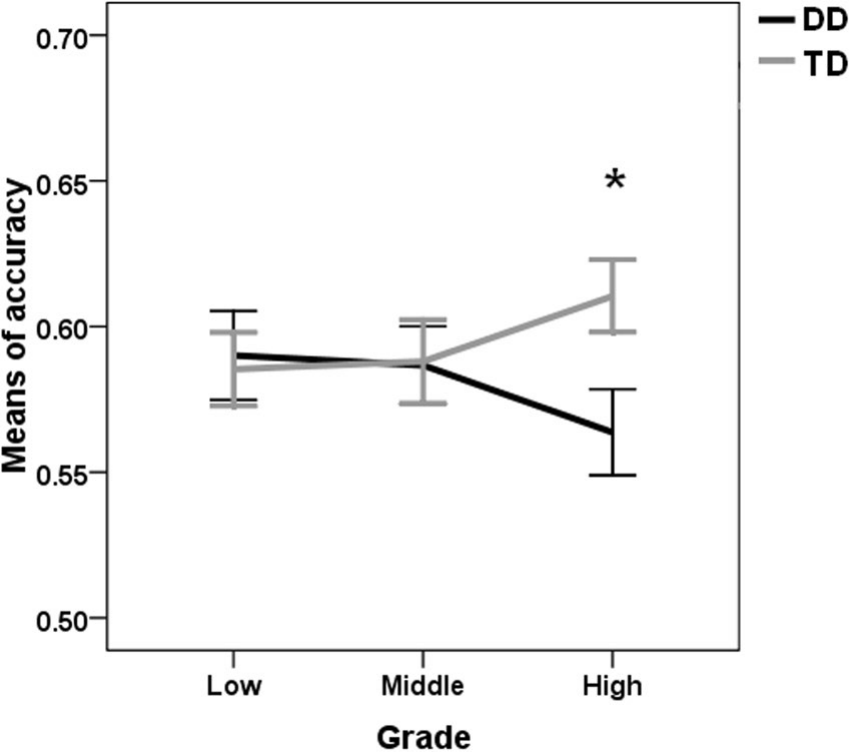
Group comparisons of the accuracy of visual 1-back tasks under each condition of eccentricity and grade. DD, children with developmental dyslexia; TD, typically developing children. *p < 0.05.

Moreover, the group × grade interaction was marginally significant [F(2, 105) = 2.59, p = 0.08, η^2^ = 0.05]. Simple effect analyses showed that the group effect was only significant in the high grade [F(1, 33) = 5.80, p = 0.02, η^2^ = 0.15] but not in the low or middle grades [low grade: F(1, 38) = 0.06, p = 0.81, η^2^ = 0.002; middle grade: F(1, 34) = 0.004, p = 0.95, η^2^ = 0.01]. In detail, the dyslexic children in the high grade made more errors in the visual 1-back task than the age-matched normal readers (Fig. 1). The grade effect was not significant for either dyslexic [F(1, 54) = 0.96, p = 0.39, η^2^ = 0.03] or normal [F(1, 51) = 1.08, p = 0.35, η^2^ = 0.04] readers.

### D-prime values

The results of the ANOVA for d-prime values showed that the main effect of the position was significant [F(4, 320) = 10.67, p < 0.001, η^2^ = 0.12]. Post hoc analyses showed that the d-prime values in the 3^rd^ position of a string was higher than that in the other four positions (ps < 0.1), and the d-prime values in the 2^nd^ position was higher than that in the 4^th^ and 5^th^ positions (ps < 0.05), and there was no any other significant comparisons (ps > 0.1).

A deviance analysis^39^ was adopted to select individuals who had deficits in their visual attention span. Because significant effects were primarily present in the analysis based on accuracy, a deviance analysis was conducted on the relevant accuracy data. Individuals were classified with a VAS impairment if their mean accuracy on the visual 1-back task was lower than the one-tail 95% confidence limit of the control group (1.65 standard deviations) after the controls with extreme scores were removed from the sample. According to the above method^39^, we calculated the cut-off accuracy for identifying VAS-impaired individuals as follows: 0.501 for the low grade, 0.491 for the middle grade, and 0.541 for the high grade. Individuals with an accuracy lower than the cut-off values, which meant poorer performance on the visual 1-back task, would be classified as having a VAS deficit. Finally, 2 of 20 children with dyslexia from the low grade (10%), 1 of 19 dyslexic readers in the middle grade (5.26%), and 7 of 18 dyslexic children in the high grade (38.89%) were identified as having an impaired VAS. For typically developing children, only 1 of 17 middle graders (5.88%) and 1 of 17 high graders (5.88%) showed VAS dysfunction.

### Analyses based on the developmental trajectories method

It has been suggested that analyses regarding group-matching design may not be applicable for examining a development pattern when a wide age range is employed^40^. To explore the development of the visual attention span in Chinese children, especially the dyslexic readers, the developmental trajectory method^40,41^ was adopted. This method, which is based on a growth model, attempted to construct a linear function linking performance with age on a specific task, such as VAS skill, and then to examine whether this function differed between typically developing children and dyslexic readers within a wide age range. The relevant results might indicate a distinction between developmental delay and atypical development in the visual attention span. If the analyses based on the developmental trajectories suggested that the development of the VAS skill was ‘delayed-prime, then the individuals with dyslexia should reach the same end point as the typical population, as would be the case for a maturation or sufficient practice. However, if the trajectories analysis suggested that the VAS development is ‘atypical’, then the dyslexic readers might never reach the endpoint achieved by the typically developing population.

In reference to previous literature^40,41^, we submitted a comparison of both the chronological age (CA) and reading level (RL) to the developmental trajectories analyses. For CA comparisons, the measures in poor readers were classified as ‘delayed-prime compared to the typically developing readers when a significant main effect of the group, or a significant age × group interaction, was demonstrated, or if both were present. The trajectories of dyslexic readers were classified as ‘atypical’ when measures and increasing CA were not linearly related for the dyslexic readers, but they were linearly related for the typically developing children. Regarding the RL comparisons, the trajectories were classified as ‘atypical’ when the task performance and increasing RL were linearly related for only one of the dyslexic and control groups but not for the other group, such as when they were linearly related for only the dyslexics instead of the normal readers. More details concerning the classification procedure and decision trees were provided in a study by Kuppen *et al*.^40^.

**Figure 2:**
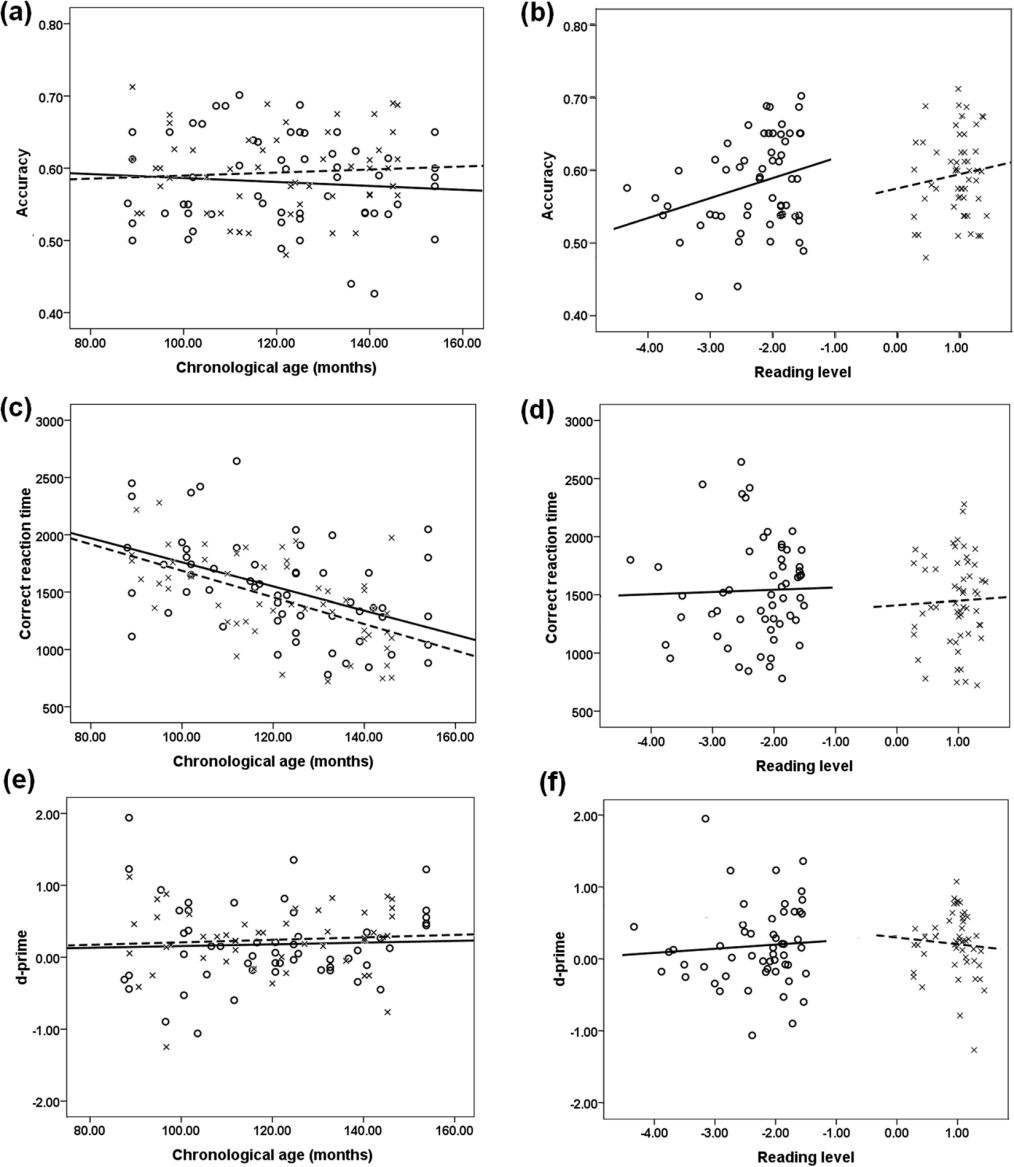
Performance at the visual 1-back task against chronological age (**a**,**c**, and **e**) and reading age (**b**,**d**, and **f**). The relevant performance at the visual attention task included the accuracy (**a**,**b**), reaction time (**c**,**d**), and d-prime values (**e**,**f**). The z values of the scores from the vocabulary test were adopted to index the reading level. The x and dashed lines represent normal readers; the o and continuous line represent the children with dyslexia.

For accuracy in the visual 1-back task (Fig. 2a,b), the task performance for both the children with dyslexia and the age-matched normal readers did not show a linear relationship with the CA, and thus the dyslexic readers were judged as showing typical developmental trajectories in the accuracy of a visual attention task. Regarding the RL comparison, normal readers showed nonlinear function, but this was not the case for the dyslexics. The trajectories of dyslexic readers were then classified as ‘atypical’.

For correct reaction times in the visual 1-back task (Fig. 2c,d), the children with dyslexia were classified as showing a delayed developmental trajectory. There was no significant main effect of the group in analyses based on CA, revealing that their trajectory did not significantly differ from that of the normal readers. The RL analysis demonstrated nonlinear relationship between the task performance and RL for either the dyslexic or normal readers.

For the d-prime values in the visual 1-back task (Fig. 2e,f), there were no significant effects. Table 2 displays the linear functions in all cases and the trajectory classification.

**Table 2:** Summary of trajectory outcomes.

Measures	CA or RA	Classification procedure			Overall trajectory classification
		Decision 1	Decision 2	Decision 3	
ACC	CA	Measures linear with CA in DDs? NO. R^2^ = 0.01, F(1,55) = 0.38, p = 0.54.	Measures linear with CA in NRs? NO. R^2^ = 0.01, F(1,52) = 0.25, p = 0.62.		NS
	RL	Measures linear with RL in DDs? YES. Y = 0.03 × + 0.64, R^2^ = 0.09, F(1,55) = 5.24, p = 0.03.	Measures linear with RL in NRs? NO. R^2^ = 0.01, F(1,52) = 0.68, p = 0.42.		Atypical
RTs	CA	Measures linear with CA in DDs? YES. Y = −10.503 × + 2809.772, R^2^ = 0.22, F(1,55) = 15.03, p < 0.001.	Main effect of group? NO. F(1, 108) = 2.06, p = 0.15.	Interaction? YES. F(2,108) = 19.67, P < 0.001.	Delay
	RL	Measures linear with RL in DDs? NO. R^2^ = 0.01, F(1,55) = 0.05, p = 0.82.	Measures linear with RL in NRs? NO. R^2^ = 0.02, F(1,55) = 0.05, p = 0.81.		NS
d-prime	CA	Measures linear with CA in DDs? NO. R^2^ = 0.04, F(1,55) = 0.09, p = 0.77.	Measures linear with CA in NRs? NO. R^2^ = 0.07, F(1,52) = 0.26, p = 0.61.		NS
	RL	Measures linear with RL in DDs? NO. R^2^ = 0.07, F(1,55) = 1.26, p = 0.61.	Measures linear with RL in NRs? NO. R^2^ = 0.07, F(1,55) = 0.21, p = 0.65.		NS

Note. CA, chronological age; RL, reading level. DD, children with dyslexia; NR, age-matched normal readers. NS, non-significant. The present study adopted the z values of the scores in the vocabulary test to index reading age.

### Correlations between the visual attention span and reading skills

Moreover, partial correlation analyses were applied to examine the relationship between performance in the visual 1-back task and reading skills within the dyslexic and normal readers of each grade, where nonverbal intelligence and participant age were controlled. Referring to previous research^42^, we separately converted the accuracy, correct reaction time, and d-prime values in the visual 1-back task from each group to Z scores, and we adopted the sum of the three types of Z scores as the measurement of the visual attention span. These Z scores were submitted to correlation analysis and further regression analysis. A Bonferroni correction was adopted for multiple comparisons. The significance was determined by using a p value of 0.0125 (0.5/4), and marginal significance was declared at a p value of 0.025 (0.1/4) after Bonferroni correction.

**Figure 3:**
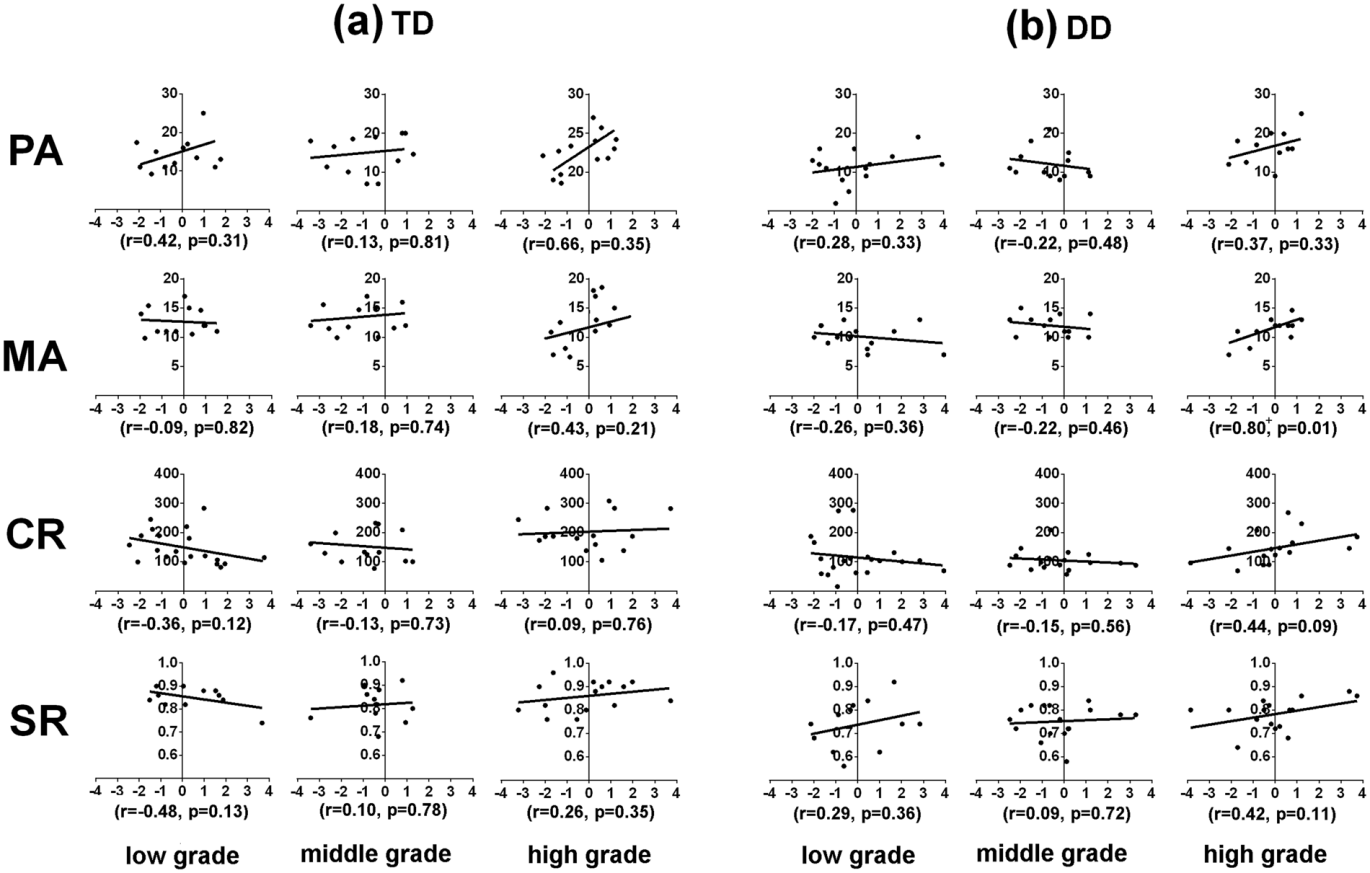
The scatter plots of the relationship between Z scores in the visual 1-back task and Chinese reading skills for normal readers and dyslexic readers in each grade. PA, phonological awareness; MA, morphological awareness. CR, character reading; SR, sentence reading. DD, children with dyslexia; TD, typically developing children. An analysis of partial correlation was conducted, in which the non-verbal intelligence and participant age were controlled. The significance was determined using a p value of 0.0125 (0.5/4), and marginal significance was declared at a p value of 0.025 (0.1/4) after Bonferroni correction. + , p < 0.025.

For normal readers (Fig. 3a), the results showed no significant correlations in any grade. For children with dyslexia (Fig. 3b), the Z scores of the visual 1-back task were marginally significantly correlated with the scores on the morphological awareness test for children in the high grade [r = 0.80, p = 0.01].

(Figure 3)

### Regression analyses for the predictive power of the visual attention span on Chinese reading

To explore the unique contribution of the visual attention span to reading skills in Chinese children from each grade, a hierarchical regression analysis was separately conducted. First, the participant ages and nonverbal intelligence were entered into the regression equation. Then, scores of phonological and morphological awareness tests were entered. Finally, the Z scores of the visual 1-back task were entered into the equation as an independent variable related to the visual attention span (Table 3). Scores for the reading test in the single-character and sentence levels were regarded as the dependent variables. The results of the regression analysis showed that VAS was a significant and independent predictor of Chinese reading only for the dyslexic readers from the high grades, and VAS accounted for a significant 45.5% of the variance in predicting reading speed at the single-character level [F(1,14) = 39.86, p = 0.008]. Additionally, we separately examined the unique contribution of phonological awareness and morphological awareness to Chinese reading. The results showed that morphological awareness could account for a significant 23.1% of variance in the prediction of speed in single-character reading for the dyslexic readers from high grades [F(1,14) = 20.25, p = 0.02]. The details of the relevant results are shown in Table 3.

**Table 3:** Hierarchical regression analyses estimating the predictive power of visual attention span, phonological awareness and morphological awareness at each grade on reading performance of the single-character and sentence levels after controlling for differences in non-verbal IQ and age.

Factors	*R*^2^ change					
	Low grade		Middle grade		High grade	
	DD (N = 20)	NR (N = 20)	DD (N = 19)	NR (N = 17)	DD (N = 18)	NR (N = 17)
Dependent variable: Reading performance in single-character level						
1.Age/Non-verbal IQ	0.055	0.447	0.219	0.642	0.373	0.807
2.PA/MA	0.111	0.025	0.110	0.289	0.138	0.193
3. VAS	0.024	0.248	0.042	0.069	0.455*	0
2. VAS/MA	0.012	0.015	0	0.185	0.569	0.193
3. PA	0.122	0.428	0.152	0.173	0.024	0
2. VAS/PA	0.130	0.441	0.077	0.045	0.362	0.193
3. MA	0.005	0.001	0.075	0.214	0.231*	0
Dependent variable: Reading performance in sentence level						
1. Age/Non-verbal IQ	0.071	0.999**	0.102	0.367	0.108	0.986
2. PA/MA	0.552	0.001	0.082	0.624	0.259	0.014
3. VAS	0.377	0	0.063	0.009	0.330	0
2. VAS/MA	0.208	0	0.003	0.485	0.427	0.014
3. PA	0.271	0	0.141	0.148	0.163	0
2. VAS/PA	0.347	0	0.071	0.072	0.355	0.014
3. MA	0.282	0	0.074	0.261	0.235	0

Note. DD, dyslexic readers; NR, normal readers. VAS, visual attention span, which represents mean accuracy in the visual 1-back task. PA, phonological awareness; MA, morphological awareness. *p < 0.05.

## Discussion

The present study investigated the developmental pattern of the visual attention span in Chinese children with developmental dyslexia by using the visual 1-back task to measure the visual attention span, in which non-verbal stimuli and no verbal response were adopted. The relevant results showed that only the dyslexic readers in the high grades, but not in the low or middle grades, exhibited lower accuracy in the visual 1-back task compared to the age-matched normal readers, revealing a developmental increase in their VAS impairment. The development trajectories of the visual attention span in Chinese children with dyslexia seemed to exhibit an atypical pattern in the analysis based on the accuracy of the visual attention task, and they seemed to exhibit a delayed pattern in the analysis on the reaction time of the visual 1-back task. The correlation analysis showed that the visual attention span was associated with morphological awareness only for the dyslexic readers from high grades. Regression analyses further exhibited that both VAS skills and morphological awareness separately and significantly contributed to single-character reading for higher-grade students with dyslexia. These findings regarding age changes revealed a developmental increasing trend in the relationship between VAS skill and reading (dis)ability.

The deficit in the visual attention span in Chinese dyslexics was absent in the low and middle grades, but it was remarkably present in the high grades of primary schools. The developmental differences could provide a possible explanation for the conflicting findings in the previous literature^36^. Mi *et al*.^36^ did not find significant differences in visual attention spans between Chinese dyslexic children and age-matched normal readers who were selected from the 2^nd^ to 5^th^ grades. Considering that VAS dysfunction was only significant in the high grades, the large range of participant ages in Mi *et al*.’s study might have been affected by the developmental changes relating to the visual attention span. Specifically, the similar capacity of the visual attention span between the dyslexic and normal readers from low/middle grades would reduce the group difference in total.

Moreover, the developmental increasing trend in the VAS impairment from the present study was consistent with the prediction based on the languages with deep orthography mentioned in the introduction section. That is, relevant findings in alphabetic languages with deep orthography suggested that the visual attention span seemed to contribute more to reading skills with age^12^. With the enhancement of reading experiences in Chinese, there was a developmental transition from relying on reading strategies of character-by-character spelling through the mediation of Pinyin to depending on direct mapping between visual forms to relevant semantics with a whole lexical route^37,43^. Because the visual attention span has been suggested to exert a more significant influence on the global coarse-grain strategy^29^ and visual-semantic processing^37,43^, it could thus be proposed that the VAS skills may be more highly required for reading processes in higher grades than during the early stages of reading development. Correspondingly, the group differences in VAS skills between the dyslexic readers and age-matched normal readers might be more obviously exhibited among higher graders compared to beginning readers. The current results based on the individual data provide support for the above inference. The proportion of VAS-impaired individuals among the dyslexics was larger in the high grades compared to the children from the low and middle grades. Moreover, the present results based on correlation analysis and regression analysis suggest that the VAS impairment in the dyslexics from high grades may directly exert some influence on Chinese reading (especially at the single-character level), such as on rapid global processing of the visual forms of several Chinese characters in foveal viewing. Additionally, this visual dysfunction might hinder morphological awareness (i.e., visual-semantic mapping) and in turn exhibit a relation to Chinese reading. Additionally, although it has been suggested that visual-spatial attention might play a role in creating visual images of auditory stimuli^35^, and there is a globally addressed route in Chinese orthographic-to-phonological mapping that is different from letter-by-letter spelling strategy based on GPC rules in alphabetic languages, the VAS ability in the current cohort in the low-grade children with dyslexia was not found to be correlated with their reading skills or phonological awareness. This finding revealed the lesser involvement of VAS in the early stages of Chinese reading development and supported the statement by Bosse *et al*.^10^ in which the VAS impairment of dyslexic readers might account for their reading disability independent of their phonological problems.

According to the developmental pattern of visual attention spans in Chinese children with dyslexia, it is necessary to explore whether the deficit of visual attention span in dyslexics revealed an atypical development or if it only lagged behind the age-matched normal readers. The present study used the developmental trajectories method to address this issue. The accuracy in the visual 1-back task generally showed an atypical pattern of development for children with dyslexia. This result revealed that the current cohort of dyslexic readers exhibited a different trajectory in their VAS development compared to the typically developing children, suggesting an atypical but not simply delayed mechanism in their VAS development. To some extent, this finding provided evidence for the hypothesis that there is a visual attention span deficit in dyslexia as proposed by Bosse *et al*.^10^, revealing the VAS impairment irrespective of language transparency. However, the trajectory analyses of the reaction times in the visual 1-back task showed a delayed pattern of development for children with dyslexia, with a parallel developmental course relative to normal readers over chronological age. These inconsistent results might be related to the complex processes involved in the reaction time. The selective reaction time in the present study might consist of the processes of visually coding the target figure, the search and retrieval from the relevant resources in short-term memory, decision making, selections between different keys, pressing the corresponding keys, etc. Some of the components in the selective reaction time may significantly develop with age, such as visual coding, key-pressing, and decision-making^44,45^, which could have covered up the developmental pattern in the visual attention span. By contrast, the data relating to the accuracy in the visual 1-back task might be more suitable for reflecting the capacity of the visual attention span. Nonetheless, future studies should use longitudinal designs (e.g., a follow-up study, or a training study) to clarify whether the underlying mechanism of VAS development in dyslexia is atypical or not.

In summary, the present study found that the deficit of a visual attention span in Chinese children with dyslexia was only present in high grades of primary school but not in the low or middle grades. The developmental increase in this impairment revealed a modulation from the orthographic transparency (i.e., deep orthography) of reading development in Chinese. Moreover, the developmental trajectory analysis suggested that the development of a visual attention span in Chinese dyslexics seemed to show an atypical pattern. This finding provides support for the hypothesis that there is a visual attention span deficit from a developmental aspect, and it has implications for reading disability remediation. Additionally, the correlation and regression analyses showed that VAS impairment might affect Chinese reading both in a direct way and through the mediation of morphological processing. However, it is worth mentioning that the results of the trajectory analysis were primarily based on accuracy in the visual 1-back task, and the sample size for correlation and regression analyses was small. Therefore, we should consider the current results as suggestive rather than conclusive. In fact, more studies should be conducted to arrive at a clear conclusion.

## Methods

### Participants

Fifty-seven Chinese children with developmental dyslexia (17 girls) and fifty-four age-matched normal readers (16 girls) were selected among 2392 primary school students from the 2^nd^ to 6^th^ grades (with a prevalence of 2.38%). Given that the grades in the primary school can be divided into three levels (i.e., low/middle/high grades)^46,47^, the current participants were divided into three groups according to their grades to explore the developmental pattern of VAS in Chinese-dyslexic readers from the cross-sectional aspect. Namely, children from the 2^nd^ and 3^rd^ grades were regarded as low graders; children from the 4^th^ grade were regarded as middle graders; and children from the 5^th^ and 6^th^ grades were high graders. All the participants had normal or corrected-to-normal vision without ophthalmologic or neurological abnormalities. No participant suffered from ADHD, as judged by the Chinese Classification of Mental Disorder 3 (CCMD-3). Detailed information for each group is presented in Table 1. The research project was approved by the Research Ethics Committee of Capital Normal University. Written informed consent was obtained from the parents and teachers. The methods were performed in accordance with the relevant guidelines and regulations.

### Psychometric tasks administered to identify dyslexics

Referring to relevant literature^3,48–50^, two tasks are widely used to identify children with developmental dyslexia in mainland China were adopted.

#### Raven’s Standard Progressive Matrices (RSPM)

The RSPM is a standardized test of nonverbal intelligence with five sets of twelve items^51^. There was a target matrix with one missing part in each item. The children were required to complete the patterns by selecting from six to eight alternatives. The difficulty increases as the test progresses. The raw score was the number of correct choices, and the standardized score was converted from the raw score based on the Chinese norms established by Zhang and Wang^51^.

#### A standardized written vocabulary test

This Chinese Character Recognition Test was adopted to measure the participant reading skills^52^. In this test, the participants were asked to write down a compound word based on a written target morpheme provided on the sheet. For example, the written target Chinese character might be “’’ (pronounced /qiu2/, meaning ball (the number here refers to tone); the participant’s task is to write another morpheme next to the target to form a real two-character (or multiple-character) word. A child responded correctly if he/she wrote, for example, “’’ (/zu2 qiu2/, which means football), or ““ (/pai1 pi2 qiu2/, which means bouncing the ball), or if they wrote another genuine word that included the target morpheme “’’. The characters are divided into 10 groups based on their reading difficulty. Each correct response was given one point. The score for each group of characters was calculated by multiplying the total points by the corresponding coefficient of difficulty. The final score for each participant was the sum of the sub-scores for all 10 character groups, which revealed the estimation of the participant’s vocabulary size.

The inclusion criteria for the dyslexics were that nonverbal intelligence was in the normal range (i.e., the standard score of RSPM was greater than that in the relevant 5th percentile), while the score on the written vocabulary was at least 1.5 standard deviations below the average score of the same-grade children. In this way, 57 children with dyslexia were identified, and 54 normal readers of the same chronological age were selected as a control group.

Moreover, to make the identification of the dyslexic readers more reliable, two reading tests were conducted at the single character and sentence levels by referring to the relevant literature^53^. The details of the reading tests were as follows. A *single character level-*a paper-and-pencil lexical decision task^37^ was adopted to examine the reading fluency at the single-character level. Children were presented with a list of 400 Chinese characters intermixed with 13 non-characters. The split-half reliability was 0.93. Participants were required to read the items aloud and to cross out the non-characters within a time limit of one minute. At the end of this test, participants were asked to mark the last item they read. The score consisted of the number of items read minus the number of errors, in which the errors were non-characters that were not identified as well as real characters that were incorrectly crossed out. The *sentence level*-a sentence verification task was utilized to assess the reading fluency at the sentence level. The split-half reliability was 0.85. A total of 54 sentences were constructed (4 for the practice session and the rest of the 50 sentences were used in the formal test). The sentences were all about simple facts, and the length of each sentence varied from seven to twenty-two characters. Half of the sentences were true, and the other half were false. This test was presented using a Dell laptop. The participants were seated approximately 50 cm from the computer screen. Within each trial, a fixation point was displayed at the center of the screen for 500 ms, and then a target of a complete sentence appeared. The participants were instructed to read the sentence aloud as quickly and accurately as possible and to press the space bar once they had finished reading the sentence. After pressing the space bar, a judgement was followed, in which the participants were required to press different keys to judge the veracity of the sentence, with ‘f’ for false and ‘j’ for true. The accuracy of the veracity judgement was recorded.

Dyslexic readers from all grades showed poorer performance than the age-matched controls did in both levels of reading tests (ps < 0.05), revealing possible impairments in reading ability, which was consistent with the previous findings^54,55^. Table 1 presents the results of the psychometric testing.

### Reading-related tasks

Reading strategies might change with development in Chinese, from relying on orthographic-to-phonological mapping to depending on visual-semantic processing^32^. Accordingly, the present study adopted two reading-related skill assessments, that is, phonological awareness (i.e., reflecting the orthographic-to-phonological mapping) and morphological awareness (i.e., reflecting visual-semantic processing), to explore the modulation of the visual attention span from reading development in Chinese. The order of these tests was randomized across participants. The details of each test are noted below.

#### Phonological awareness

The phonological awareness test used in the present study was designed by Qian *et al*.^49^. The split-half reliability was 0.92. This test measured sensitivity to the onsets, rimes and tones of spoken Chinese monosyllabic words. An onset and a rime make up of a Chinese syllable, which may be pronounced in up to four different tones (the 1^st^ tone: high level tone, the 2^nd^ tone: rising tone, the 3^rd^ tone: falling-rising tone, and the 4^th^ tone: falling tone). For example, for the monosyllable “bang4”, its onset is “b” at the beginning, and its rime is “ang”, and it sounds with the fall tone (i.e., the 4^th^ tone). An odd-one-out paradigm was adopted in this test, and there were three types of oddity, onset, rime, and lexical tone. There were 30 trials in the phonological test, with 10 trials for each type of oddity. Detailed information regarding the presentation format of this test was stated in Zhao *et al*.^56^. The accuracy was recorded. If a participant made a correct response for one trial, then he/she was awarded 1 point. The total score was 30.

#### Morphological awareness

To reveal the visual-semantic processing, the morphological awareness was assessed with a paper-and-pencil test of homograph awareness. The split-half reliability was 0.94. During this task, twenty pairs of two-character words in Chinese were presented. One character in the two words of each pair was in common. The participants were required to judge whether this overlapping character had the same meaning in the two words or not. For example, the pair of words “”(/hua1yuan2/, meaning “flower garden”) and “”(/hua1qian2/, meaning “spend money”) had the same character “”/hua1/, which exhibited different meanings in the two words with the meaning “flower” in the former one and “spend” in the latter. There were 2 practice pairs and 20 test pairs. A correct response was awarded 1 point. The total score was 20.

The dyslexics exhibited lower accuracy than the age-matched controls did in tests of phonological awareness [low grade: t_38_ = 2.22, p = 0.04; middle grade: t_34_ = 1.99, p = 0.049; and high grade: t_33_ = 2.45, p = 0.03], and morphological awareness [low grade: t_38_ = 2.86, p = 0.01; middle grade: t_34_ = 2.58, p = 0.02; high grade: t_33_ = 1.85, p = 0.08, marginally significant].

### Visual tests

#### Test of visual attention span

**Figure 4:**
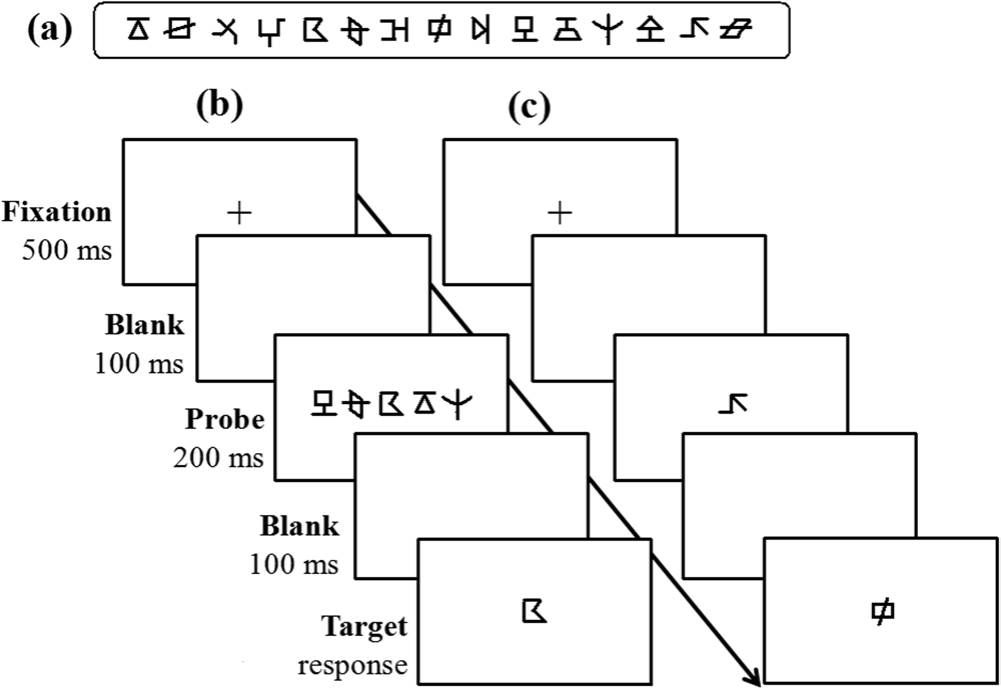
The presentation format of each trial in the visual 1-back task and the control task. (**a**) shows all 15 figures used in the visual 1-back task and the control task. (**b**) displays the procedure for the visual 1-back task. In each trial, a 500-ms fixation point was first presented at the center of the screen, followed by a 100-ms blank and then a probe of the five-figure string centered on the fixation, for 200 ms. The string was followed by a 100-ms blank, and finally, the target of a single figure appeared below or above (each for half of the trials) the median horizontal line. The participants were required to press different keys to judge whether the target figure was present in the above string or not. The letter (**c**) shows the presentation format of each trial in the control task for single figure recognition. The procedure was generally similar to that in the visual 1-back task, except that the probe comprised only one figure instead of a five-figure string. The participants were asked to judge whether the target was the same as the probe or not, by pressing different keys.

Referring to relevant studies^37,38^, the visual 1-back paradigm was adopted here to measure the visual attention span skills with non-verbal stimuli and no verbal response. The test-retest reliability was 0.81. The stimuli in this test were 15 figures (Fig. 4a). A list of 80 five-figure strings was created using the 15 figures. No string included the same figure twice. They were presented in black on a white screen with E-prime 1.1 on a Dell laptop. The display resolution was set at 1024 × 768 with a monitor refresh rate of 75 Hz. The visual angle of the strings was 7.9° × 0.8° at a distance of 50 cm. The center-to-center distance between each adjacent figure was 1.7°. The presentation format of each trial was consistent with the study by Zhao *et al*.^37^. In each trial (Fig. 4b), a 500-ms fixation was first presented in the screen center, and then there was a 100-ms blank, which was followed by a probe of the five-figure string centering on the screen for 200 ms. The string was replaced by a 100-ms blank, and finally, the target of a single figure appeared below or above (half of the trials) the median horizontal line. Participants were asked to press the Z key as quickly and accurately as possible when the target figure was present in the above string and to press the B key when it was absent. The target figure was replaced by a blank screen after the response. The blank screen lasted for a random interval (from 1000 ms to 1500 ms) between successive trials. The 80 trials were presented randomly and included 50 target-present trials and 30 target-absent trials. The response time and accuracy were recorded. The d-prime scores were also computed based on the accuracy means in each position of the five-figure strings, which was a bias-free estimate of task sensitivity^58^. All the participants were tested individually in a quiet room. At the beginning of the experiment, the experimenter explained the procedure in detail from a standard script. The test trials were preceded by 10 practice trials.

#### The control task

A recognition task using a single figure (Fig. 4c) was designed to assess whether the participants had sufficient efficiency at identifying an individual figure and to examine whether their short-term memory exerted an influence on performance in the visual 1-back task. The stimuli were the 15 figures mentioned above (Fig. 4a). The presentation procedure for each trial was generally similar to that used in the visual 1-back task, except that the probe consisted of one figure rather than a five-figure string. The participants were required to judge whether the probe and target were the same. ‘Same’ decisions were indicated by pressing the Z key and ‘different’ decisions by pressing the B key. This experiment was programmed using E-prime 1.1. The reaction time and accuracy were recorded, and the d-prime scores were also computed based on accuracy means. The formal experiment consisted of 48 trials, with 30 trials for the ‘same’ decisions (twice for each figure) and 18 trials for the ‘different’ decisions. The proportion of the two types of responses (same vs. different; 5:3) was the same as that in the visual 1-back task.

### Data availability statement

The data in the current study are available upon request.
